# Study protocol for a randomized controlled trial to evaluate the effectiveness of an artificial intelligence-based health education accurately linking system based on traditional Chinese medicine body constitution in patients with chronic disease multimorbidity

**DOI:** 10.3389/fpubh.2026.1773974

**Published:** 2026-02-27

**Authors:** Qingqing Liu, Yuqi Yang, Yi Hu, Xiaohua Yang, Suxiang Liu, Rantong Bao, Yibo Wu

**Affiliations:** 1 Department of Nursing, the Fourth Affiliated Hospital of School of Medicine, and International School of Medicine, International Institutes of Medicine, Zhejiang University, Yiwu, China; 2 School of Nursing, Henan University of Science and Technology, Luoyang, Henan, China; 3 School of Nursing, Shandong Second Medical University, WeiFang, China; 4 Center for Clinical Epidemiology Research, The Affiliated Hospital of InnerMongolia Medical University, Hohhot, China

**Keywords:** AI-HEALS, chronic disease multimorbidity, mobilehealth (mHealth), randomized controlled trial (RCT), traditional Chinese medicine body constitution

## Abstract

**Background:**

The prevalence of chronic disease multimorbidity among Chinese adults is high and presents a serious public health challenge. Personalized and accessible lifestyle interventions are key to improving long-term outcomes. Existing mobile health technologies often deliver generalized content, offering high accessibility but overlooking the potential of individualized interventions informed by Traditional Chinese Medicine (TCM) body constitution. TCM emphasizes identification of constitution types to develop more targeted and comprehensive personalized lifestyle guidance. Therefore, we developed the Artificial Intelligence–Health Education and Lifestyle Support system based on TCM body constitution (AI-HEALS) to explore its feasibility and assess its preliminary efficacy in improving clinical outcomes, enhancing self-management behaviors among patients with chronic disease multimorbidity.

**Methods:**

This study adopts a three-arm parallel randomized controlled trial design. A total of 207 patients with hypertension, type 2 diabetes, and/or hyperlipidemia will be recruited and randomized in a 1:1:1 ratio into a routine chronic disease management group, a generalized AI-HEALS intervention group, or a TCM-based AI-HEALS intervention group. The routine management group will receive standard chronic disease care only. AI-HEALS is deployed via a WeChat Mini Program and features automated health-education content delivery, digital diaries for data logging, and intelligent Q&A functions. The backend houses a knowledge base covering chronic disease multimorbidity and TCM constitution-based wellness. The generalized AI-HEALS group will receive health-education content targeting chronic disease multimorbidity. The TCM-based AI-HEALS group will first complete a constitution assessment through the Mini Program, after which the system will deliver personalized health-management content tailored to their constitution type, drawing from the combined knowledge base of chronic disease multimorbidity and TCM constitution wellness. The intervention will last 3 months with follow-ups to month 6. The primary outcome is the change in systolic blood pressure from baseline to the end of intervention. Secondary outcomes include blood glucose and lipid levels, chronic-disease self-management behaviors, among others. Changes in TCM constitution classification will be treated as an exploratory outcome.

**Discussion:**

This study integrates TCM constitution theory with the AI-HEALS system to provide an innovative, personalized, and accessible management strategy for chronic disease multimorbidity, and seeks to explore the value of combining traditional medical theory with modern information technology. This protocol describes a randomized controlled trial designed to generate preliminary evidence on effectiveness.

**Clinical trial registration:**

https://www.chictr.org.cn/index.html, identifier ChiCTR2600118267

## 1 Background

Approximately one quarter of Chinese adults are affected by chronic disease multimorbidity, with hypertension combined with dyslipidemia or diabetes representing the most common patterns of coexistence (1). Hypertension, diabetes, and dyslipidemia are independent major risk factors for cardiovascular disease (2), and when they occur together, the risk of cardiovascular events increases multiplicatively (3). The resulting cardiovascular and cerebrovascular diseases not only severely threaten patients’ quality of life but also impose a substantial economic burden (4, 5). In 2020, total hospitalization costs for cardiovascular and cerebrovascular diseases in China reached 270.901 billion yuan, of which hypertension and diabetes accounted for 13.260 billion yuan and 31.641 billion yuan, respectively. Such multimorbidity has become a serious challenge to public health and socioeconomic development (6).

Individuals’ lifestyle choices and behaviors are deeply rooted in their surrounding sociocultural environment (7). In this context, Traditional Chinese Medicine (TCM) constitution theory provides a time-tested framework for understanding individual differences. It aims to reveal the unique body constitution formed through interactions between innate endowment and acquired environmental influences, and to elucidate its intrinsic relationship with health and disease (8, 9). More targeted and personalized intervention strategies can be developed by accurately identifying one’s constitution type (10, 11). Individuals with phlegm-dampness, blood stasis, or Yin-deficiency constitutions have significantly higher risks of metabolic syndrome compared with those of a more balanced constitution, and these constitution types are positively correlated with key metabolic indicators (12). Interventions such as “strengthening the spleen and resolving dampness” for phlegm-dampness constitution can improve body weight and glycemic control (13), while “nourishing Yin and moistening dryness” for Yin-deficiency constitution may help regulate lipid metabolism (14). Despite differences in specific treatment methods, these approaches commonly involve TCM constitution–based lifestyle-modification guidance—including diet, exercise, daily routine, emotional regulation, and supportive use of Chinese herbal medicine (14). Such interventions have been shown to enhance chronic-disease self-management (15), reduce pharmaceutical medication dependence (16), lower chronic-disease susceptibility (17), and improve quality of life (15). However, traditional healthcare models rely on infrequent, episodic consultations that are costly and limited in continuity and accessibility (18), greatly restricting their scalability. Providing continuous, comprehensive, accessible, and personalized lifestyle interventions for patients with chronic multimorbidity (19) can effectively enhance their self-management capabilities (20) and subsequently improve clinical outcomes in diabetes, hypertension, and dyslipidemia (21). The Constitution in Chinese Medicine Questionnaire (CCMQ), issued by the China Assocition of Chinese Medicine, has demonstared good reliability and validity and is a commonly used of Chinese Medicine, has demonstrated good reliability and validity and is a commonly used instrument for constitution identification (22). While an individual’s constitution exhibits a degree of stability, it can evolve over the life course due to influences from lifestyle and internal or external environments (23). However, most existing studies on constitution are cross-sectional or case–control investigations with small sample sizes, which can suggest but cannot substantiate causal relationships between constitution-based interventions and health outcomes (24). This study is designed as an evidence-building effort to contribute more robust empirical evidence in this area.

The widespread adoption of mobile health technologies has improved the accessibility of healthcare services (25), and numerous studies have demonstrated their positive effects on managing chronic disease indicators such as blood pressure and blood glucose (26–29). However, a detailed examination of these studies (30, 31) reveals that mobile health interventions for chronic diseases typically rely on standard management supplemented with app-based health-education messages. Such approaches overlook the substantial individual differences among patients with chronic multimorbidity, making it difficult to achieve precise, root-cause-oriented regulation tailored to each person (32, 33). Integrating the *individualization* principles of TCM body constitution with the *scalability* and *empowerment* of mobile health technologies may offer a new model of personalized, patient-centered health management.

In response, our research team previously developed the Artificial Intelligence–based Health Education Accurately Linking System (AI-HEALS). AI-HEALS is constructed on the Multitheoretical Model (MTM) framework for health behavior change and comprises two modules—AI-Chatbot and HEALS— which are delivered via the WeChat Mini-Program. The AI-Chatbot module is designed to emulate an “integrative Chinese–Western medicine clinical expert.” It utilizes natural language processing (NLP) technology. Having been pre-trained and fine-tuned on authoritative medical literature (including guidelines, textbooks, and research papers), and iteratively refined through multiple rounds of expert feedback. This enables the module to conduct interactive question-answering with context-aware understanding and semantic reasoning. It incorporates data-driven machine-learning components that support continuous learning and performance enhancement. The HEALS module is responsible for pushing personalized health content to users. It does not rely on machine-learning or adaptive algorithms, but instead on a rule-based decision logic combined with user-behavior data analysis for content matching. Specifically, the system builds a basic interest profile by recording user interaction (e.g., sections clicked, reading duration, and time) and selects appropriate content from an expert-curated knowledge base according to predefined rules (e.g., disease type, intervention stage, TCM constitution classification). Therefore, HEALS functions as a structured, rule-driven decision-support component, with personalization derived from its rule engine and layered content design. Collectively, the system features automated delivery of health-education messages, digital diary functions, and intelligent question-and-answer capabilities. Its backend contains a knowledge base—updated every 3 months—covering chronic disease multimorbidity and TCM constitution-based wellness guidance. A core innovation is the embedded TCM body-constitution assessment module: after users complete the constitution evaluation via the WeChat Mini Program, the system dynamically delivers personalized health-management content based on the TCM constitution wellness knowledge base, while the intelligent Q&A module provides real-time responses to patient inquiries. The aims of this study are as follows:

1. To evaluate the effectiveness of the AI-HEALS system—particularly its TCM-adapted version—compared with routine management and generalized mobile health interventions in improving clinical outcomes among patients with chronic disease multimorbidity.
2. To assess the effectiveness of the system in enhancing chronic-disease self-management behaviors.

## 2 Methods

### 2.1 Study design

This study will adopt a single-blind, three-arm parallel randomized controlled trial design. Participants will be recruited from a Class A secondary hospital in Zhejiang Province, China. All enrolled patients will receive standard chronic disease management in accordance with *Expert consensus on standardized diagnosis and treatment of coexisting hypertension, hyperglycemia, and hyperlipidemia in China (2023 edition)* (3). The intervention period will last for 3 months, followed by continued observation until month six to assess the sustainability of short-term effects. The three groups differ in the additional interventions provided:

- Control Group (CG): Routine chronic disease management only.
- Universal AI-HEALS Group (UG): Routine management plus access to the AI-HEALS WeChat Mini Program (universal version).
- TCM-Adapted AI-HEALS Group (TG): Routine management plus access to the AI-HEALS WeChat Mini Program (TCM-adapted version).

**Figure 1:**
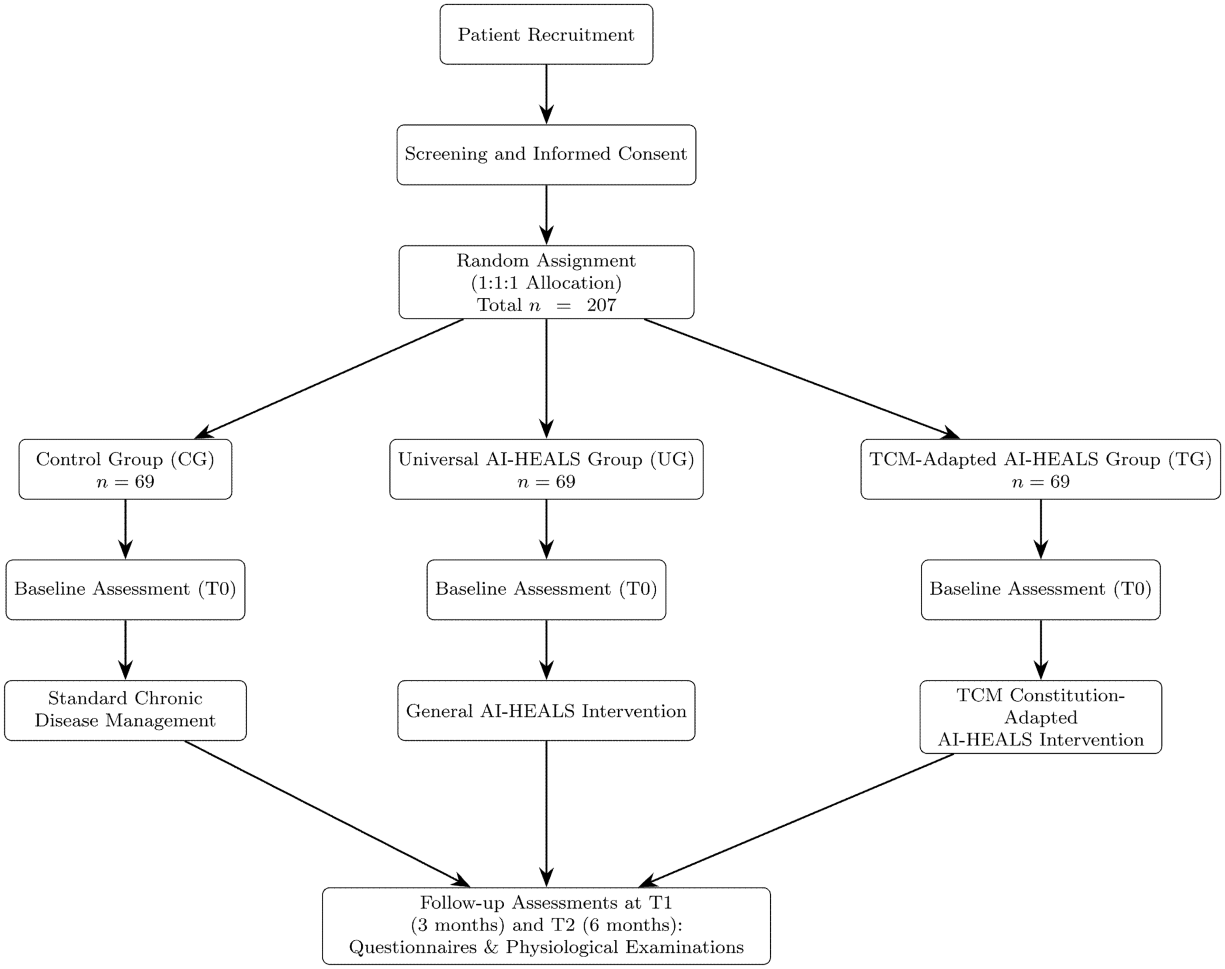
Flow chart of patient recruitment and study implementation.

The study flow and group allocation are shown in Figure 1.

### 2.2 Participants

#### 2.2.1 Recruitment and sample size

This study plans to use a consecutive enrollment strategy between February 2026 and December 2026. Specialized personnel from the hospital Information Department (not involved in this study) will conduct an initial screening of anonymized data from the electronic health record system to identify patients who meet the diagnostic criteria for multimorbidity. Subsequently, research assistants will independently review the data in pairs based on predetermined inclusion and exclusion criteria. Once eligibility is confirmed, independent recruitment staff will contact potential participants using compliant contact information recorded in the hospital system and invite them to join the study after obtaining informed consent.

The sample size calculation is based on the primary outcome (systolic blood pressure). According to previous similar research (34–36), the optimistic but clinically significant effect size hypothesis was adopted. The expected systolic blood pressure after 3 months of intervention is set as follows: 138.2 mmHg for the routine health education group, 132.0 mmHg for the universal AI group, and 127.0 mmHg for the TCM-constitution–based AI group. The common inter-group standard deviation (*σ*) is set at 16.3 mmHg. Using G*Power 3.1 software with one-way ANOVA (F tests), two-sided *α* = 0.05, and power (1 − *β*) = 90%, the required sample size is 55 participants per group, totaling 165 participants. Considering an estimated 20% dropout rate, the final planned recruitment is 207 participants, or 69 per group. If the true intervention effect size is smaller than the assumed, the statistical power of this study will be correspondingly reduced, and the results should be interpreted with caution and regarded as preliminary exploratory evidence.

#### 2.2.2 Inclusion and exclusion criteria

##### 2.2.2.1 Inclusion criteria

Age ≥ 18 years; confirmed diagnosis of hypertension, type 2 diabetes, and/or hyperlipidemia as multimorbidity; Proficient in using a smartphone and capable of operating applications such as WeChat; Independent in daily living, mentally competent, and able to communicate clearly; able to complete questionnaires and willingness to participate.

##### 2.2.2.2 Exclusion criteria

Presence of severe impairment in vital organs such as the heart, liver, or kidneys; Received radiotherapy or chemotherapy within the past 6 months; pregnant or breastfeeding women; current participation in other clinical studies.

### 2.3 Randomization and blinding

#### 2.3.1 Randomization

A computer-generated randomization sequence will be used. The sequence will be generated in Excel by an independent statistical expert with no conflict of interest before participant enrollment. To ensure allocation concealment, a study coordinator (not involved in recruitment, enrollment, or intervention delivery) will manage the randomization process. After screening, included participants will receive a unique non-sequential identifier (e.g., “ID-001”), linked only to the allocation information and not to intervention details. The study coordinator will assign participants to groups based on this identifier and provide group assignment only to the intervention implementation team, ensuring that other personnel (including recruiters and outcome assessors) remain blinded before the intervention.

#### 2.3.2 Blinding

This study adopts a single-blind design. Due to the nature of the interventions, participants and intervention providers cannot be blinded. However, research assistants responsible for collecting outcome data and statisticians conducting the final data analysis will remain blinded to group allocation. Research assistants will not know participants’ group assignments during clinical measurement and questionnaire collection. Statisticians will analyze de-identified datasets and will not access files containing allocation information before the analysis is completed.

### 2.4 Interventions

All three groups will undergo a 3-month intervention period.

#### 2.4.1 Control group (CG)

Participants will receive routine chronic disease management, including outpatient follow-up, health education, medication guidance, and monitoring of blood pressure, blood glucose, and blood lipids. They will not access the AI-HEALS platform, nor undergo TCM constitution assessment.

#### 2.4.2 Universal AI-HEALS Group (UG)

In addition to routine chronic disease management, this group will receive an AI-HEALS-based intelligent health education intervention delivered through a WeChat Mini Program. The intervention operates via three core modules: rule base construction, article recommendation, and an intelligent Q&A system. The platform implements a combined model of “menu-driven self-intervention and targeted content delivery “to provide continuous, individualized, and adaptive health education and behavior support for patients with hypertension, hyperlipidemia, and diabetes multimorbidity”. The platform also includes the following ancillary and adherence-support features: Daily reminders: The system automatically sends daily tips on diet, exercise, and daily routines. A check-in function allows participants to log completion of recommended behaviors and record blood pressure and glucose measurements; Additional support: Research staff will provide telephone assistance to each participant to ensure smooth operation of the platform throughout the intervention period. High-adherence criterion: Participants will be classified as highly adherent if they log into the system on at least 3 days per week during the intervention phase.

#### 2.4.3 TCM-Adapted AI-HEALS Group (TG)

This group receives routine care and uses AI-HEALS enhanced with a TCM-expert-curated knowledge base to deliver constitution-based personalized interventions.

- Platform initialization and constitution profiling: During the first use of -AI-HEALS, participants will complete TCM constitution identification using the relevant scale, along with baseline self-report questionnaires and entry of key health indicators (e.g., blood pressure, blood glucose, lipid profiles)
- Personalized health profiling: Based on the 30-item simplified Constitution in Chinese Medicine Questionnaire (CCMQ-30) (37), the system automatically identifies the participant’s constitution type. By integrating disease status, lifestyle habits, and symptomatic manifestations, it generates a personalized “health profile” to inform subsequent tailored interventions.
- Interactive support: Users can actively ask questions through text or voice input and receive individualized health consultation and management advice. The platform homepage also provides structured menu access, allowing participants to browse health topics of interest.

All intervention content is designed according to TCM constitution and covers core domains: dietary adjustment, exercise guidance, daily routine regulation, emotional regulation, and related health-preservation advice.

Introduction to TCM constitution is provided in Appendix 1 in Supplementary Material.

TCM-based health education materials are provided in Supplementary material 1.

### 2.5 Outcomes

Data will be collected at baseline (T0), month 3 (T1), and month 6 (T2).

#### 2.5.1 Primary outcome

Change in systolic blood pressure from baseline to month 3 (T1). Systolic blood pressure (SBP) is selected due to its continuous, independent, and direct positive association with cardiovascular risk (2), and strong evidence that effective SBP control reduces cardiovascular events and all-cause mortality (38).

#### 2.5.2 Key secondary outcomes

- Physiological indicators: HbA1c, blood fat, BMI, diastolic pressure.
- Self-management behavior modification: ability to self-manage chronic disease, medication adherence, physical activity, level of engagement, Self-Regulation of Eating Behavior.
- Quality of life and psychological indicators: quality of life, anxiety, depression, self-efficacy, Perceived Social Support.

#### 2.5.3 Exploratory outcomes

- TCM constitution indicators: constitution type transformation.

Timing of measurements is shown in Table 1.

**Table 1:** Schedule of evaluations.

Study period	Recruitment	Intervention	Follow-up
Informed consent	√		
Demographics	√		
Physiological indicators	√	√	√
Medical history and medication use	√	√	√
CCMQ-30	√	√	√
CDSMS	√	√	√
PHQ-4	√	√	√
EQ-5D-5L	√	√	√
ARMS-7	√	√	√
NGSES	√	√	√
PSSS	√	√	√
SREBQ	√	√	√

CCMQ-30, Constitution in Chinese Medicine Questionnaire–30-Item Short Form; CDSMS, Chronic Disease Self-Management scale; PHQ-4, the abbreviated Patient Health Questionnaire-4; EQ-5D-5L, EuroQol-5 Dimensions 5 Levels; ARMS-7, Adherence to Refills and Medications Scale-7; NGSES, New General Self-Efficacy Scale; PSSS, The Perceived Social Support Scale; SREBQ, The Self-Regulation of Eating Behavior Questionnaire.

### 2.6 Collected information

We will collect sociodemographic information, including: age, sex, marital status, type of medical insurance, education level, employment status, household income, smoking history, drinking history, etc.

#### 2.6.1 Blood pressure measurement

Blood pressure will be measured using hospital-certified and fully calibrated electronic devices. During measurement, participants will sit quietly for at least 5 min, relax their body, maintain the upper arm at heart level, and remain seated. After 30–60 s, the measurement will be repeated, and the average of two readings will be used.

#### 2.6.2 BMI measurement

These will be measured twice using hospital-certified and fully calibrated instruments. BMI will be calculated as weight (kg) divided by height (m).

#### 2.6.3 Questionnaires

1. Traditional Chinese Medicine (TCM) constitution identification: Participants will be assessed using the *Constitution in Chinese Medicine Questionnaire – 30-Item Short Form* (CCMQ-30) developed by Professor Wang Qi (37), which evaluates an individual’s TCM constitution type over the past year. It consists of 9 subscales (Balanced constitution and 8 biased constitutions: Qi-deficiency, Yang-deficiency, Yin-deficiency, Phlegm-dampness, Damp-heat, Blood-stasis, Qi-stagnation, and Special diathesis), totaling 30 items. In Chinese population samples, the Cronbach’s *α* coefficients for the CCMQ-30 range from 0.51 to 0.75.
2. Chronic disease self-management: Assessed using the *Chronic Disease Self-Management Study Measures* (39), developed by Lorig et al. and introduced into China by Fu Dongbo et al. (40) for use in hypertensive patients. The scale includes three dimensions: cognitive symptom management practices, exercise, and communication with physicians.
3. Quality of life: Assessed using the *EQ-5D-5L* scale (41), developed by the EuroQol Group, covering five dimensions: mobility, self-care, usual activities, pain/discomfort, and anxiety/depression.
4. Psychological status: Assessed using the *PHQ-4* ultra-brief anxiety and depression screening tool (42), developed by Kroenke et al. in 2009. It contains two depression items and two anxiety items.
5. Medication adherence: Assessed using the *Adherence to Refills and Medications Scale* (ARMS-7) (43), developed by Kripalani et al. Used to evaluate medication-taking behaviors and refill adherence. It includes 7 items across two subscales: medication-taking (Items 1–4) and refill behaviors (Items 5–7).
6. Self-efficacy: Assessed using the *New General Self-Efficacy Scale (3-item version)* (NGEES) (44), developed by Chen et al. in 2001. It is a unidimensional scale with 8 items, used to measure overall confidence in dealing with new tasks or adversity.
7. Perceived Social Support: Assessed using the *Perceived Social Support Scale-Short Form (PSSS-SF)* (45), developed by Zimet et al. in 1988. The PSSS-Short Form (PSSS-SF) is a brief version derived from the original scale by selecting three highly representative items.
8. Self-Regulation of Eating Behavior: Assessed using *The Self-Regulation of Eating Behavior Questionnaire (SREBQ)* (46), It is a 5-item, single-dimensional scale designed to assess an individual’s capacity for dietary self-regulation.

#### 2.6.4 Process indicators

System usage frequency, function usage preferences, and intervention satisfaction assessed through interviews.

### 2.7 Statistical analysis

Statistical analyses will be performed using SPSS 26.0 (or R). Continuous variables with normal distribution will be presented as mean ± standard deviation; non-normally distributed variables as median (interquartile range).

All primary and key secondary analyses will adhere to the intention-to-treat principle. To evaluate the effect of the intervention on health outcomes, under the assumption of data missing at random, a linear mixed-effects model will be applied. Time, group, and their interaction will be included as fixed effects, and a random intercept will be added for each participant to control for baseline heterogeneity. Baseline values and age will be included as covariates.

Between-group differences at each time point will be estimated using the EMMEANS package to compute estimated marginal means (EMMs), followed by adjusted pairwise comparisons (e.g., Tukey multiple comparison tests). To assess overall changes from baseline to the end of follow-up, model-estimated baseline-adjusted marginal means at the endpoint will be compared among the three groups. The family-wise error rate for primary and key secondary outcomes will be controlled using the Bonferroni method. A *p*-value < 0.05 will be considered statistically significant.

Sensitivity analyses will be conducted to assess the robustness of the findings:

- Adherence analyses: To examine the impact of intervention adherence, a per-protocol analysis will be performed. Participants meeting the pre-defined criterion for high adherence will be included, and results will be compared with the primary intention-to-treat analysis.
- Missing Date: To evaluate the robustness of findings against departures from the missing-at-random assumption, a worst-case scenario analysis will be performed. In this analysis, missing values in the intervention groups will be imputed under the assumption of clinical deterioration, whereas missing values in the control group will be imputed under the assumption of improvement.
- Exploratory analyses will be conducted to generate hypotheses for future research. These will include:
- Subgroup analyses: Treatment effect heterogeneity will be explored across subgroups defined by baseline characteristics (e.g., age, sex, having all three conditions) by adding relevant interaction terms to the linear mixed-effects model.
- Analysis by Comorbidity Pattern: This analysis will specifically examine whether intervention effects differ among participants with varying multimorbidity patterns (e.g., hypertension + diabetes vs. Hypertension +hyperlipidemia vs. all three conditions).

Results from all Exploratory Analysis will be interpreted with caution due to reduced statistical power. For exploratory outcomes (TCM constitution type transformation), the focus will be on estimating effect sizes and confidence intervals rather than hypothesis testing. When multiple *p*-values need to be combined, Stouffer’s Z-score method will be utilized.

## 3 Research management

This study has been approved and is overseen by the Ethics Committee of the Pujiang County Traditional Chinese Medicine Hospital. A Data Monitoring Committee (DMC), consisting of two independent members with no conflicts of interest, will supervise the trial and conduct safety and progress assessments every 6 months. All research staff must complete standard operation procedure (SOP) and ethics training.

### 3.1 Informed consent

All participants will be fully informed of the study details during recruitment and will sign written informed consent.

### 3.2 Adverse event handling

All adverse events (AEs) and serious adverse events (SAEs) must be reported to the Ethics Committee and the DMC within 24 h. In the event of an SAE, the intervention for the affected participant will be immediately discontinued, and appropriate medical care will be provided.

### 3.3 Data security

Data will be double-entered and cross-checked by two independent staff members. Electronic data will be stored on password-protected encrypted servers with strictly restricted access. Unblinding will occur only after study completion and final data analysis, under supervision from the DMC and conducted only by the principal investigator and statistician.

## 4 Discussion

TCM constitution, as an integrated attribute reflecting an individual’s physiological, psychological, and environmental adaptability, embodies the principle of personalized treatment based on “treatment tailored to the individual.” Its core lies in providing precision health management based on constitution differentiation.

Given the substantial public healthcare burden caused by chronic multimorbidity and the urgent need for individualized management among patients, mobile health technologies—which overcome temporal and spatial constraints of healthcare services—offer an important tool for modernizing TCM constitution theory.

AI-HEALS integrates artificial intelligence with mobile health technology, enabling automated and precise personalized interventions through its knowledge base, intelligent Q&A, and other modules. By deeply combining TCM constitution theory with AI-HEALS, the study aims to build an intervention model of “constitution identification → precise content delivery → real-time feedback,” addressing multimorbid patients’ needs for individualized, accessible, and continuous management.

Using standardized tools to identify constitution types, the system tailors health-education content by linking TCM health-preservation principles with current multimorbidity management guidelines. Its intelligent Q&A module provides immediate responses to patient questions, thereby enhancing engagement and adherence.

Prior studies have shown that TCM constitution-based interventions can improve metabolic outcomes and quality of life among patients with chronic diseases (47, 48), and AI-powered mobile health tools have proven effective in managing single chronic conditions (49, 50). Yet, for individuals with multimorbidity, the combined application of TCM constitutional theory and AI-HEALS remains insufficiently studied. There is a lack of constitution-tailored multimorbidity management strategies, and the potential synergistic benefits of integrating these two approaches are still unclear.

Through a randomized controlled trial, this study aims to generate evidence on the feasibility and effectiveness of a precision, integrative (TCM–biomedicine) management model for patients with multimorbidity.

This study has the following limitations: Due to the nature of the intervention, a double-blind or triple-blind design could not be implemented, which may introduce expectation bias in participants. Particularly, this study has the following limitations: Due to the nature of the intervention, a double-blind or triple-blind design could not be implemented, which may introduce expectation bias among participants. In particular, in the TCM Constitution Intervention Group, participants might experience the Hawthorne effect due to their perception of the intervention as “more personalized.” To mitigate such effects, we strictly standardized the push frequency, reminder mode, interactive features, and follow-up across the three groups, with only the content generation logic being set to reflect the intended differences. Additionally, the primary outcome measures were objective clinical indicators to minimize subjective reporting bias.
