# Supplementary material for: Study protocol for a randomized controlled trial to evaluate the effectiveness of an artificial intelligence-based health education accurately linking system based on traditional Chinese medicine body constitution in patients with chronic disease multimorbidity
Source: Front Public Health. 2026 Feb 27;14:1773974. doi: 10.3389/fpubh.2026.1773974 (PMC12982435; doi:10.3389/fpubh.2026.1773974)
Supplement: Supplementary file 1 [file Supplementary_file_1.docx]

Examples of TCM Constitution–Based Health Education Content

| **Constitution Type** | **Main Features (Plain-language version)** | **Dietary Recommendations** | **Daily Routine** | **Exercise** | **Emotional Well-being** |
| --- | --- | --- | --- | --- | --- |
| **Balanced (Healthy Type)** | Rosy complexion, good energy, good appetite, stable sleep, strong adaptability | Balanced diet; grains, vegetables, fruits, meat, and eggs; avoid picky eating | Regular schedule; early to bed and early to rise; adapt to seasonal changes | Exercise if you enjoy it; if not, avoid long sitting; walk 30 min/day | Stay calm and optimistic |
| **Qi-deficient (Easily fatigued)** | Fatigue, weak voice, spontaneous sweating, prone to colds | Eat Chinese yam (Shan Yao), red dates, lotus seeds, millet, chicken; avoid raw/cold/bitter foods | Avoid staying up late or overexertion; keep warm, especially during seasonal transitions | Gentle exercises: Tai Chi, Baduanjin (Qigong), walking; avoid strenuous exercise | Reduce worry; relaxation techniques; get more sunlight |
| **Yang-deficient (Cold type)** | Aversion to cold, cold hands/feet, preference for warm drinks, low energy, loose stools | Warm foods: ginger, lamb, longan fruit, walnuts; avoid cold drinks, watermelon, mung beans | Keep warm, especially lower back and feet; avoid going out early on winter mornings | Activities in sunlight: jogging, brisk walking; avoid heavy sweating | Stay positive; join warm, lively activities |
| **Yin-deficient (Internal-heat type)** | Warm palms/soles, dry mouth/throat, night sweats, insomnia/dream-disturbed sleep | Eat snow ear fungus (Tremella mushroom) , lily bulb, pear, duck, black sesame; avoid spicy, BBQ, alcohol | No staying up late; sleep before 11 pm; room should be cool and properly humid | Calm, gentle activities: yoga, Tai Chi, walking; avoid heavy sweating | Reduce anger and anxiety; quiet the mind; listen to soothing music |
| **Phlegm-damp (Obesity type)** | Overweight body, big belly, oily face, thick/greasy tongue coating, snoring | Light diet; winter melon, coix seed, red beans, radish; avoid fatty foods, sweets, alcohol | Keep environment dry and ventilated; avoid long sitting/lying | Aerobic exercise: brisk walking, swimming, cycling; at least 5 times/week | Develop hobbies, socialize more, avoid being cooped up at home |
| **Damp-heat (Acne type)** | Oily face, acne, bitter taste, bad breath, dark yellow urine, sticky stools | Eat mung beans, bitter melon, celery, cucumber; avoid fried foods, spicy foods, alcohol | Keep skin clean; change clothes frequently; avoid hot/humid environments (steam rooms/saunas) | Moderate sweating is good, but shower promptly after | Control temper; remain calm; avoid irritability |
| **Blood stasis (Dark complexion)** | Dark complexion, purplish lips, dark circles, menstrual clots | Eat hawthorn, black fungus (Auricularia mushroom), rose tea, onion; avoid cold foods | Keep warm, especially during menstruation and winter; avoid cold exposure | Stretching, dance, Tai Chi to promote circulation | Maintain a  cheerful mood; avoid long-term suppression or anger |
| **Qi stagnation (Sensitive type)** | Low mood, sighing, chest tightness, poor sleep, easily tense | Eat citrus, finger citron (Fo Shou), mint, wheat; avoid strong tea and coffee | Keep a regular lifestyle; create a warm and comfortable home | Participate in group activities: singing, dancing, ball games | Make friends, express feelings, develop hobbies (painting, gardening) |
| **Special/Inherent (Atopic type)** | Prone to allergies (rhinitis, asthma, hives), sensitive to smells/pollen | Avoid known allergens (e.g., seafood, mango); eat light and bland foods | Keep indoor space clean; reduce pets; avoid going out in pollen season | Moderate exercise to enhance immunity ; avoid polluted or peak-allergy periods | Maintain stable emotions; avoid fright or excessive stress |
