# Supplementary material for: Study protocol for a randomized controlled trial to evaluate the effectiveness of an artificial intelligence-based health education accurately linking system based on traditional Chinese medicine body constitution in patients with chronic disease multimorbidity
Source: Front Public Health. 2026 Feb 27;14:1773974. doi: 10.3389/fpubh.2026.1773974 (PMC12982435; doi:10.3389/fpubh.2026.1773974)
Supplement: Supplementary file 2 [file Supplementary_file_2.docx]

# ****Appendix 1: TCM Constitution****

## ****1. What is TCM Constitution?****

TCM constitution refers to the comprehensive and relatively stable intrinsic characteristics formed in the human life process, based on congenital endowment and postnatal influences. It encompasses an individual’s morphological structure, physiological functions, and psychological state. It represents the unique traits that humans develop during growth and development to adapt to natural and social environments.

## ****2. Common Types of TCM Constitution****

There are **9 subtypes**:

- Balanced constitution (平和质)
- Qi-deficient constitution (气虚质)
- Yang-deficient constitution (阳虚质)
- Yin-deficient constitution (阴虚质)
- Phlegm-dampness constitution (痰湿质)
- Damp-heat constitution (湿热质)
- Blood stasis constitution (血瘀质)
- Qi stagnation constitution (气郁质)
- Special/inherited constitution (特禀质)
